# A highly redundant BAC library of Atlantic salmon (*Salmo salar*): an important tool for salmon projects

**DOI:** 10.1186/1471-2164-6-50

**Published:** 2005-04-04

**Authors:** Jim Thorsen, Baoli Zhu, Eirik Frengen, Kazutoyo Osoegawa, Pieter J de Jong, Ben F Koop, William S Davidson, Bjørn Høyheim

**Affiliations:** 1 Department of Basic Sciences and Aquatic Medicine, Section of Genetics, Norwegian School of Veterinary Science, P.O box 8146 Dep, N-0033 Oslo, Norway; 2 BACPAC Resources, Children's Hospital Oakland Research Institute, Oakland, CA, USA; 3 Biotechnology Centre of Oslo, University of Oslo, Oslo, Norway; 4 Institute of Medical Genetics, University of Oslo, P.O. Box 1036, Blindern, N-0315 Oslo, Norway; 5 Centre for Biomedical Research, Department of Biology, University of Victoria, Victoria, BC, V8P 5C2, Canada; 6 Department of Molecular Biology and Biochemistry, Simon Fraser University, Burnaby, BC, V5A 1S6, Canada; 7 Department of Dermatology, National Hospital, Rikshospitalet, N-0027 Oslo, Norway

## Abstract

**Background:**

As farming of Atlantic salmon is growing as an aquaculture enterprise, the need to identify the genomic mechanisms for specific traits is becoming more important in breeding and management of the animal. Traits of importance might be related to growth, disease resistance, food conversion efficiency, color or taste. To identify genomic regions responsible for specific traits, genomic large insert libraries have previously proven to be of crucial importance. These large insert libraries can be screened using gene or genetic markers in order to identify and map regions of interest. Furthermore, large-scale mapping can utilize highly redundant libraries in genome projects, and hence provide valuable data on the genome structure.

**Results:**

Here we report the construction and characterization of a highly redundant bacterial artificial chromosome (BAC) library constructed from a Norwegian aquaculture strain male of Atlantic salmon (*Salmo salar*). The library consists of a total number of 305 557 clones, in which approximately 299 000 are recombinants. The average insert size of the library is 188 kbp, representing 18-fold genome coverage. High-density filters each consisting of 18 432 clones spotted in duplicates have been produced for hybridization screening, and are publicly available [1]. To characterize the library, 15 expressed sequence tags (ESTs) derived overgos and 12 oligo sequences derived from microsatellite markers were used in hybridization screening of the complete BAC library. Secondary hybridizations with individual probes were performed for the clones detected. The BACs positive for the EST probes were fingerprinted and mapped into contigs, yielding an average of 3 contigs for each probe. Clones identified using genomic probes were PCR verified using microsatellite specific primers.

**Conclusion:**

Identification of genes and genomic regions of interest is greatly aided by the availability of the CHORI-214 Atlantic salmon BAC library. We have demonstrated the library's ability to identify specific genes and genetic markers using hybridization, PCR and fingerprinting experiments. In addition, multiple fingerprinting contigs indicated a pseudo-tetraploidity of the Atlantic salmon genome. The highly redundant CHORI-214 BAC library is expected to be an important resource for mapping and sequencing of the Atlantic salmon genome.

## Background

Atlantic salmon (*Salmo salar*) is a fish native to the basin of the North Atlantic Ocean. As farming of Atlantic salmon is growing as an agricultural enterprise, identification of genetic regions and genes responsible for economically important traits might be of importance for future agriculture of salmon.

Genome projects in farm animals aim at identifying regions of the genome responsible for traits of economic importance in order to implement this into breeding and management programs. This is for instance achieved by performing QTL studies to identify genomic regions that are linked to traits of economic importance. Genetic and physical maps, publicly available databases and cDNA libraries from various tissues are a few of the tools needed to identify genomic regions responsible for traits of interest. Currently there are two published linkage maps for Atlantic salmon [2,3] and several EST databases available to the public [4-8]. After a genomic region has been shown to be linked to a trait of economic importance, a high quality BAC library resource is crucial in the identification and functional characterization of the genetic variation.

Atlantic salmon along with all fish of the family *Salmonidae *shows residual tetraploidity after a duplication event that occurred 25–100 Myr ago [9]. However, rapid chromosome divergence has been observed between different salmonid species, involving Robertsonian changes as well as other structural rearrangements [9]. Hence, the pseudo-tetraploid state is challenging for researchers working with the Atlantic salmon genome.

Here we describe the construction and characterization of a highly redundant BAC library of Atlantic salmon. To our knowledge, this is the first reported Atlantic salmon BAC library, which will be an important tool for constructing physical maps and in the identification and sequencing of regions of the Salmon genome. The large insert BAC clones will be useful as to provide better understanding of the pseudo-tetraploid state, identify regions of interest to the aquaculture industry as well as to the basic science community.

## Results and discussion

**Figure 1:**
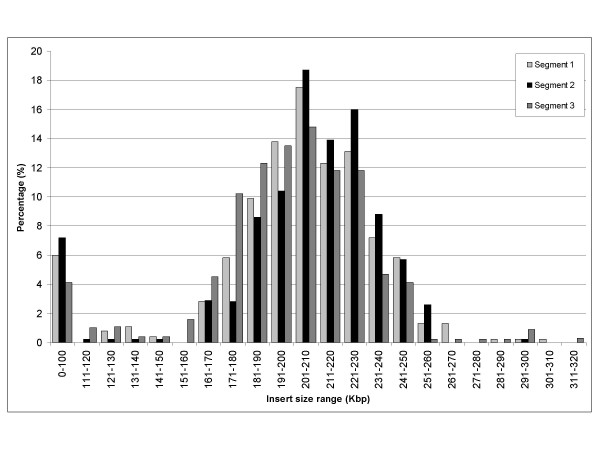
**Size distribution of BAC clone insert sizes in the CHORI-214 library. **A total of 249, 218, and 220 clones from each of the segment 1–3 was digested with NotI and analyzed by PFGE. The horizontal axis refers to the range of insert sizes, and the vertical axis indicates percentage of clones corresponding to each size range in each of the three segments. The grey tones represent the segments as indicated

The current BAC library, CHORI 214, consists of three segments. The average insert size was estimated based upon analysis of NotI digested DNA isolated from 249, 218, and 220 clones from segment 1, 2 and 3 respectively. The distribution of insert sizes is presented in Figure 1. Based on these results, the average insert size was estimated to be 188 kbp. With a total of approximately 299 000 clones the BAC library provides 18.8 haploid genome equivalents (see Table 3) given a genome size of 3.27 pg (6.55 pg/N) [10] or similar 3.10 pg at the Genome Size Database [11].

**Table 3:** Details of the three segments of the BAC library

Segment	1	2	3	All segments
Plate numbers	1–288	289–576	577–816	1–816
Plate count	288	288	240	816
Empty wells	1982 (1.79%)	2830 (2.56%)	2975 (3.23%)	7787 (2.49%)
Non-recombinant clones	0	5	4	9
Non-insert clones ^1^	1.2% ^2^	2.8% ^3^	2.3% ^4^	2.0%
Recombinant clones ^5^	~107 000	~105 000	~87 000	~299 000
Average insert size	189 Kbp	190 Kbp	186 Kbp	188 Kbp
Genomic coverage	6.8X	6.6X	5.4X	18.8X

Genomic DNA obtained from Salmon sperm was partially digested with EcoRI restriction enzyme and cloned in pTARBAC2.1 vector.

^1 ^Estimated numbers for each segment is based upon NotI digestion.

^2 ^3 out of 249 clones were identified as non-insert clones

^3 ^6 out of 218 clones were identified as non-insert clones

^4 ^5 out of 220 clones were identified as non-insert clones

^5 ^Recombinant clones have been estimated from the total well number subtracting non-insert clones, non-recombinant clones and empty wells.

In the construction of the library, great care was made to estimate the amount of enzymes to be used for the digestion and dephosphorylation of the vector. Excessive amounts of either enzyme showed an increase in ligation products containing non-insert clones with a smaller vector fragment. These products are possibly due to star activity and deletion in the SacB gene or its promoter region, causing the clones to survive on media containing sucrose. The number of non-insert clones were estimated to represent about 2% of the library based upon the results from the NotI digestion analysis (see Table 3). Nine non-recombinant clones, which contain the complete BAC vector only, were detected in the entire library by hybridization using pUC19 DNA onto the filter sets. Reducing the frequency of non-recombinant and non-insert clones is important as these clones can easily dominate the clone collection of the library due to reduced transformation efficiency of large DNA fragments in *E. coli*. High frequency of non-insert and non-recombinant clones will increase the cost for storage and utilization of the library. In addition, large scale random fingerprinting and sequencing will have a significant reduced cost using libraries with low frequency of non-insert and non-recombinant clones.

To further characterize the library, a set of 34 oligo probes generated from sequence flanking known non-linked microsatellite markers was selected to be used in screening of the 17 BAC library colony filters. These filters contain the complete BAC library gridded in duplicate. In an attempt to reduce the probability of these probes to contain repeat sequences, each probe was hybridized to one BAC filter in order to check the quality of the probe as a unique marker. From these test hybridizations, in combination with Southern blots of genomic DNA digested with TaqI, PstI and MspI, 12 probes were characterized as unique with 0 to 4 signals onto a single BAC filter and single bands onto the genomic filters (results not shown). The three enzymes TaqI, PstI and MspI were chosen because of a high frequency of restriction sites for these enzymes in the salmon genome. As many as 20 of the 32 probes yielded several hundreds to thousands of positive double signals onto one BAC filter and multiple bands or smear onto the genomic southern blots indicating that the probes contained repetitive sequence elements.

The 12 probes, which were assumed not to contain repeat elements based on the initial hybridization results, were hybridized as one pool to the filters and resulted in the identification of 396 positive BAC clones. To identify the relationships between the probes and the clones, a second hybridization experiment was performed for each probe individually onto new small nylon filters containing all 396 clones. Out of the 396 BAC clones identified in the primary screening, 233 were verified in these secondary screening experiments (Table 1). In addition, all the clones that were not assigned to a probe in the second hybridization experiments, possible false positives, were analyzed individually in PCR experiments with all the microsatellite PCR primer pairs, resulting in the assignment of an additional 91 BAC clones to the microsatellite markers (Table 1). See additional file 1 for a complete data set of BAC clones linked to each microsatellite marker.

**Table 1:** Number of BAC clones detected after hybridization and PCR verification of probes flanking microsatellites

GenBank accession no.	Mic.sat. clone name	No. of clones observed in secondary hybridisation	Verified by PCR	Positive PCR product on unassigned clones	Total no. of clones verified by PCR
AF256671	BHMS-175	28	28	7	35
AF256676	BHMS-189	24	21	5	26
AF256678	BHMS-201	9	9	4	13
AF256741	BHMS-289	19	18	5	23
AF256698	BHMS-304	19	18	6	24
AF256748	BHMS-330	12	12	1	13
AF256746	BHMS-349	23	23	4	27
AF256719	BHMS-429	42	42	29	71
AF256693	BHMS-278	8	*	*	*
AF256848	BHMS-255	24	#	#	#
AF256750	BHMS-337	21	5	29	34
AF256714	BHMS-396	4	3	1	4
Total number of clones:		233	179	91	270
Average		19.4	19.9	9.1	27.0

* PCR products was not observed from BAC template, and a weak smear was observed using genomic DNA

# Weak smear was observed from BAC template and genomic DNA

Furthermore, we also screened the CHORI 214 BAC library using probes specific to known expressed sequences. Hence, 15 overgos were constructed from Atlantic salmon EST sequences (Table 2). These probes were hybridized to the BAC filters as one pool, and 1203 BAC clones were verified as positive for the 15 probes after a second hybridization experiment. All the BAC clones were fingerprinted in order to provide additional information about their organization. The fingerprinting results generated multiple contigs for 14 of the probes while the clones for the last probe resulted in six single clones. Out of the 1203 clones, 160 clones were not mapped into contigs and are here referred to as singletons. The observation of singletons is either caused by clones containing small insert sizes, possible false positives or lower quality DNA loaded onto the agarose gel. The FPC software will not be able to map small insert clones into contigs using the selected cut off value. For a complete data set of BAC clones linked to each gene marker and the respective fingerprinting contigs see additional file 1.

**Table 2:** Contigs assembled from the hybridization of 15 EST derived overgo probes onto the BAC filters

					No. of BAC clones in each contig						
					
GenBank accession no.	Putative gene	No. of positive BACs identified	Singletons	No. of contigs	1	2	3	4	5	6	7
BG 934178	Elongation factor 2	79	19	3	28	18	14				
BG934439	Eukaryotic translation initiation factor 5	91	3	2	78	10					
BG935804	Ubiquitin	69	8	2	54	3					
BG934353	Phosphate transfer protein	108	14	4	47	31	8	4			
BG935917	cyclin L ania-6a	6	6	0							
AF201470	Retinal rod opsin	100	16	3	64	17	3				
AJ344158	Myostatin, isoform II	104	10	4	42	22	17	10			
BG936489	Actin related protein (P16arc)	122	10	3	51	39	20				
BG933799	AMP deaminase	76	12	5	38	7	7	4	3		
BG933794	TAR DNA-binding protein	69	11	2	35	22					
BG934675	Helicase	142	25	7	35	25	22	16	9	5	4
BE518514	Translation initiation factor 3	17	0	2	13	4					
X14305	Growth hormone	74	8	3	42	20	3				
BG935839	Collagen type I	97	7	6	37	28	12	4	4	3	
BG935084	Transducin alpha subunit	49	11	3	15	12	9				
Average:		80.2	10.7	3.3							

Contigs with 3 or more clones are shown

An average of 80 BAC clones was observed in the screening of the EST derived overgo probes, ranging from 6 to 142 BAC clones and 3 contigs on average for each of these probes. However, the results obtained using the genomic oligos resulted in an average of 19.4 clones after secondary hybridization screening, and an average of 27 clones from PCR verification (Table 1). The average observed with the genomic probes corresponds to the estimated genome coverage of 18.8 fold. Because of selection pressure one would expect duplicated expressed DNA sequences to be more preserved than non-coding sequences. Consequently, probes containing expressed sequences might more often identify the pseudo-duplicated regions (in addition to possible pseudo genes) than non-coding probes. However, using fingerprinting, these pseudo-duplicated regions containing restriction enzyme differences might still be mapped to different contigs. Observation of multiple fingerprinting contigs has previously been reported in a BAC library for Rainbow trout (*Onchorhynchus mykiss*), a member of the *Salmonidae *family, using gene and EST probes in screening of the library [12]. The authors present their results as evidence of locus duplication in Rainbow trout. Our results are in agreement with these findings [12]. Our approach, using both expressed sequences as well as non-coding sequences, might be more suited in characterization of BAC libraries of pseudo-ploidity state genomes than expressed sequences exclusively.

## Conclusion

We have constructed a BAC library from Atlantic salmon (*Salmo salar*). Based on the number of clones and the observed average insert size of 188 kb, we estimate the library to have in excess of 18-fold genome representation.

Providing a publicly available highly redundant genomic large insert library for Atlantic salmon is important for several reasons. First, genomic large insert clones can be used to construct contigs for regions of interest, fingerprinted in large scale and thus create large physical maps of the salmon genome, or be used in shotgun sequencing approaches. Secondly, utilizing one library would be important in exchange of data between researchers. The characterization reported here illustrates the usefulness of the library in identifying genomic clones and the possibility of utilizing clones in fingerprinting analysis. The very large average insert size of the clones combined with the high redundancy will provide researchers with a possibility of obtaining complete gene sequences within a single BAC clone. This might be useful for expression studies of genes and their regulatory elements. The BAC library of Atlantic salmon will be an important tool for future salmon projects and to the salmon industry.

## Methods

### Library construction

The BAC library was constructed following the protocols from [13-15] using the pTARBAC2.1 vector [16]. Transformation of the ligation products was performed using electrocompetent *E. coli *DH10B T1 phage resistant cells (ElectroMAX DH10B T1 resistant, Invitrogen). High-density replica filters were prepared as previously described [14].

### High molecular weight DNA preparation

Sperm cells were isolated from one single Atlantic salmon which was provided by Aqua Gen AS [17], and embedded in agarose plugs at a final concentration of 5 × 10^7 ^cells/ml followed by a sequence of treatments [15]. DNA used in the library construction was partially digested with EcoRI in the presence of EcoRI Methylase, and size fractionated by pulsed-field gel electrophoresis (PFGE) using a CHEF apparatus (BioRad). The size fractioned agarose gels were stored in 0.5 M EDTA until use. Electroelution procedures were used in obtaining the partially digested DNA from the gel slices [15].

### BAC vector preparation

The pTARBAC2.1 plasmid DNA was isolated using cesium chloride gradient purification [15], digested with ApaLI and EcoRI and treated with calf intestine phosphatase (CIP, from New England Biolabs) [15], and separated on 1.0% agarose CHEF gel. The vector fragment was purified from the gel as previously described [15].

### Insert size analysis

One clone from each 384 plate in the library was inoculated in LB medium containing 20 μg/ml chloramphenicol. The clones were grown for 18 hours and the BAC DNA was purified using the automated plasmid isolation machine AutoGen 960 (AutoGen). BAC DNA was digested with NotI and analyzed by PFGE [13]. Low Range PFG Marker (New England Biolabs) was used as DNA size marker. The molecular weight determination was achieved using Alpha Innotech MultiImage digital imager and AlphaEase computer software (Alpha Innotech).

### Hybridization screening

A set of twelve 36-mer oligo probes from unique genomic sequence flanking twelve different microsatellite markers were end labeled using PNK4 and hybridized as one pool onto the filters. In addition, 15 probes from Atlantic salmon EST sequences were used to design overlapping oligonucleotide probes (overgo; [18]). All probes used are presented in Table 4. The microsatellite oligos and the EST overgos were used in hybridization as two separate pools. Hybridization was carried out in tubes at 65°C overnight in Church buffer without BSA [19]. The filters were washed 4 times at 65°C each for 15 minutes using 1.5 X SSC and 0.1% SDS. Identification of positive signals was achieved by exposing the filters to either X-ray film (Agfa) or to Phosphor Image cassettes (Amersham Biosciences). All the clones identified in the screening were arrayed into new 384 plates, spotted onto nylon filters using a 384 pin tool, grown overnight on LB agar containing 20 μg/ml chloramphenicol, and processed following established procedures [19]. Each filter was hybridized with the individual probes, and the clones identified were re-arrayed in 96 well plates.

Genomic DNA was isolated using established phenol/chloroform extraction procedures [19], digested with TaqI, PstI and MspI and separated on a 1% TBE agarose gel. DNA was transferred to nylon filters using established Southern blot techniques [19].

**Table 4:** Primer sequences for EST and genomic probes

	EST probe	
	
GenBank accession no.	Overgo A primer	Overgo B primer
15844006	5'-AAGCCTGTGCTGATGATGAACAAG-3'	5'-GCAGGGCACGGTCCATCTTGTTCA-3'
15844267	5'-GTCGTTTATGTCATCCCCTCTTCT-3'	5'-GGTATCTTCTGTCTGGAGAAGAGG-3'
15845632	5'-GAAGGCATCCCTCCTGATCAGCAG-3'	5'-CAGCGAAGATCAACCTCTGCTGAT-3'
15844181	5'-TACTCCATGCAGGGACTCTGCAAG-3'	5'-CCTCATAGAAGCCAAACTTGCAGA-3'
15845745	5'-GCGACCAGCTACATTTACCAAAGC-3'	5'-ATTCCACATCACCCAGGCTTTGGT-3'
7271780	5'-GCTTCCCCATCAACTTCCTCACGC-3'	5'-TCGATGGTGACGTAGAGCGTGAGG-3'
16604728	5'-ACTGGATTATTGCCCCTAAGCGCT-3'	5'-CAGTAGTTGGCCTTGTAGCGCTTA-3'
15846317	5'-TGCCACAAGTTCATGCGCTTCATG-3'	5'-TCTCGGCTCTCATCATCATGAAGC-3'
15843627	5'-ATGTCTCCGCTCAGCAACAACAGC-3'	5'-GGTAGCTGAGGAAGAGGCTGTTGT-3'
15843622	5'-GAGCCTAAGCACAATAATAGTAGG-3'	5'-CACGATCCATCATTTGCCTACTAT-3'
15844503	5'-CCATCAAGAAGGACGAGGACGTGC-3'	5'-GGGCAGTTCTTCTTCAGCACGTCC-3'
15967287	5'-GAGCTTCCAGCTGGTGGACACTGC-3'	5'-AGTCTTCTGCGTCTTGGCAGTGTC-3'
15845667	5'-CACTTGCTTAAGCTGGGCTCTATC-3'	5'-TCCATTGGTCCTCTCCGATAGAGC-3'
15843919	5'-AAGATCCCAGGTGGGCGAGGGAAT-3'	5'-TGTGATCCCGCTGACCATTCCCTC-3'
15844912	5'-AACATCCTGCAGTCTGCTCTGGCC-3'	5'-CCATGCCTCTGATGATGGCCAGAG-3'
	PCR primers for microsatellites	
	
	Forward primer	Reverse primer

AF256671	5'-TCACATCCCTTAGCTCCC-3'	5'-CCTTTTTTGTGTCTTCAGC-3'
AF256676	5'-AAACACCCTTCCCTTCAC-3'	5'-CAATTCAGGTCAAACCAAC-3'
AF256678	5'-CCCCATGATGTGTTCTTC-3'	5'-CACAATGAGGCTTGACAC-3'
AF256741	5'-TTGAGCCATCCTCACCTC-3'	5'-CACTGGTTTGTTGTTGTTG-3'
AF256698	5'-CAGAACCGTGATCTGAAG-3'	5'-TGGACATTCTCTGGCGTC-3'
AF256748	5'-CTAGATCACTCACCCAGG-3'	5'-GTGCTTTTGGCTTATGTTAG-3'
AF256746	5'-GCTGTGATTTCTCTCTGC-3'	5'-AAAGGTGGGTCCAAGGAC-3'
AF256719	5'-CCCCTGTCAAACGTCTTC-3'	5'-AGCACACTGGATTCAAGG-3'
AF256693	5'-AGGCACAAACATGCAAGC-3'	5'-TCACCCCTGTGTCATCAC-3'
AF256848	5'-TCCAAACCTGAATCCAGG-3'	5'-TTGTAGTGAAAGCCGCTG-3'
AF256750	5'-TCCCACTGCCAACTACAG-3'	5'-GTTTAATCAAAGCATTCGCC-3'
AF256714	5'-CCTGCCATCATCCAACTC-3'	5'-TCCACACCCAACATACTC-3'
	Genomic probe sequence	

AF256671	5'-GCAGCTCAGTGACTATGACTTCTCCGGTTTCCTGTTCTCT-3'	
AF256676	5'-GCCCTAGAGATTGAAATAGCATCCTCTTTCACGCCATGCA-3'	
AF256678	5'-CTGCAAGACAGAGAACACCATGACACACAGACCTCTGGAT-3'	
AF256741	5'-AGTGAAGACCTCAACCCACAAAGGCGCTATAATCGGCAAC-3'	
AF256698	5'-TCCTGTGTATCTGCAGTCAGTTCCAGGAAATGGAGGAGCA-3'	
AF256748	5'-TGAGGGGGCTTACAAGAGGTCTTCGCTTTGCCCCAGAAAA-3'	
AF256746	5'-CACAGTTGCCAGTTGAGAGAAGAGAAAGACGTTAGGGACA-3'	
AF256719	5'-TGGCAAAGCCTAGAGAGGTTTATCTCAGCACCACATTGCA-3'	
AF256693	5'-TCACCCCTCATTCACACAATCTCCAGCTGTCACATCAAGC-3'	
AF256848	5'-TTCTCGGCTAGATCACTTGCTCTGTCTCTCTTCCCCACTC-3'	
AF256750	5'-TCCTGTAGCATGCTGACATTCTGGCAGTCAGACACACAAG-3'	
AF256714	5'-CGCTGACTTGATTTGCCTTAATGCAGTATGTGTCAACCCC-3'	

### PCR verification of positive clones

PCR primers were constructed based on the genomic sequence of the microsatellites used in the screening of the library. Cultures containing 20 μg/ml chloramphenicol and 7% glycerol of the re-arrayed clones identified by the microsatellite oligos were transferred to new 96 well plates containing PCR reaction mix. PCR reactions were carried out in 96 well plates in a total volume of 25 μl, containing 0.2 mM dNTPs (Amersham Biosciences), 1X PCR buffer (Qiagen), 7 pmol of each primer and 0.25 units of Taq DNA polymerase (Qiagen). The PCR reactions were incubated at 95°C for 3 minutes, followed by 35 cycles at 95°C for 30 seconds, annealing at 54°C for 30 seconds, and extension at 72°C for 30 seconds with a final incubation at 72°C for 10 minutes. After thermocycling, 2 μl of each PCR reaction was analyzed on a 1.5% agarose gel (ReadyGel, Amersham Biosciences).

### Fingerprinting and contig assembly

DNA from all the clones identified in the screening using the EST overgos was isolated using the AutoGen 960 (AutoGen), digested with HindIII and DNA fragments were separated in a 1.2% agarose gel using established protocols [20]. The gels were stained using SYBRGreen and scanned using a FluorImager 595 (Amersham Biosciences). Gel images were analyzed using Image 3.10 [6] and the data obtained were used with FPC v4.7 [6]. A fixed tolerance of seven, cut-off value of e-14 and a bury value of 10% was used to build contigs. To merge contigs, the cut-off value was raised to e-12 to contigs that corresponded to the individual EST sequences

## Authors' contributions

JT performed the experiments and drafted the manuscript. BZ, EF and BH provided supervision. KO, PJD, BFK, WSD and BH coordinated the project.

## Supplementary Material

Additional File 1 Probes and associated BAC clones. A list of probes and the associated BAC clones Click here for file
